# Experimental Analysis of the Influence of Heat Treatments on the Flexibility of NiTi Alloy for Endodontic Instruments Manufacturing

**DOI:** 10.3390/ma16093437

**Published:** 2023-04-28

**Authors:** Ihssen Abdelmomen, Marin Vincent, Frédéric Thiebaud, Julia Budzinski, Thierry Bastogne, Tarak Ben Zineb, Marc Engels-Deutsch

**Affiliations:** 1 Université de Lorraine, CNRS, LEM3, F-54000 Nancy, France; 2 Faculté d’Odontologie de Lorraine, Université de Lorraine, F-54000 Nancy, France; 3 CYBERnano, F-54505 Nancy, France; 4 Université de Lorraine, CNRS, CRAN, F-54000 Nancy, France

**Keywords:** endodontic files, Nickel-Titanium, shape memory alloy, phase transformation, bending, statistical design of experiments, heat-treatment

## Abstract

The flexibility of NiTi based endodontic files is improved by heat treatment, leading to lower risk of failure, ledges, and canal transportation during the preparation of curved root canals. The aim of this study is to investigate and clearly highlight the influence of every parameter of heat treatment on the flexibility of NiTi wires and thus of endodontic instruments. A full factorial Design of Experiment (DoE) and a designed bending–torsion bench following the ISO 3630-1 standard were used for this investigation. Temperature, holding time, and cooling method were selected as contributing factors, while maximum bending moment, hysteresis size, and stiffness during martensitic transformation were selected as outputs. Regression analysis was performed to estimate the relationship between contributing and output variables to assess how the experimentation fits with the model. The experimental results showed that wires heated at 425 °C for 30 min are more flexible. Moreover, heat treatment temperature is the most critical factor influencing the flexibility and hysteresis size of the NiTi wire followed by the holding time, while the cooling method has a negligible effect. The regression analysis showed that the model is effective at predicting the relationship between contributing factors, bending moment response, and hysteresis size.

## 1. Introduction

Since the introduction of Nickel-Titanium (NiTi) Shape Memory Alloys (SMAs) as the material of choice for manufacturing endodontic rotary instruments, the efficiency of root canal preparation has been significantly enhanced. This is mainly due to the properties of NiTi-SMAs such as Superelasticity and Shape Memory Effect (SME) playing a key role in the response of rotary instruments [1,2,3]. These unique properties of NiTi have led to new biomedical applications such as neurosurgery and robot-assisted surgery devices, in addition to cardiovascular devices, as well as controlled assistive and rehabilitation orthopedic devices [4].

These characteristics have also led to a reduction in treatment time, a simplification of instrumentation procedures, and an increase in predictability and effectiveness of endodontic treatments. Nevertheless, intracanal separation of NiTi rotary instruments is still a major concern for endodontists and can compromise the outcome of endodontic therapy [5]. Previous work has reported that normal and shear stresses related to bending and torsion loadings, occurring during intracanal rotation, are the two causes of rotary files fracture [6,7].

Different strategies to increase efficiency, safety, and reliability of NiTi rotary files have been proposed, by both manufacturers and scientists, to provide superior mechanical properties of endodontic files. Over the last few years, many modifications have been observed, including innovations in instrument geometric design, incorporation and hybridization of new movement strategies to drive instrumentation systems, and surface and heat treatments of NiTi alloys [8,9]. Although only NiTi-based SMAs are used in endodontic practice, other SMAs—especially Cu-based single crystals (Cu-Al-Be)—are emerging thanks to their high flexibility and biological antibacterial properties [10,11,12].

Heat treatments are generally performed during the production of NiTi wires but can also be carried out by endodontic manufacturers before, during, and after manufacturing of NiTi instruments [13]. The aim of heat treatments is to decrease the work-hardening of the SMA before its machining as well as to recover its superelasticity by releasing internal stresses caused by machining, therefore reducing dislocations, decreasing hardness, and increasing ductility [14,15,16]. Consequently, heat treatment either before or after machining is one of the fundamental approaches used to modify the crystal arrangement of the alloy and to change the phase transformation behavior of NiTi SMAs [15,16,17,18,19,20,21]. On the other hand, heat treatment of NiTi SMAs aims also to shift the transformation temperatures such as M_s_ (Martensite start), M_f_ (Martensite finish), A_s_ (Austenite start), and A_f_ (Austenite finish) in order to reduce the yield stresses values for forward and reverse start and end of martensitic transformation [22,23,24].

Superelastic conventional NiTi instruments are initially used in the austenite phase at body and buccal temperatures. During root canal preparation, the normal and shear stresses due to the curvature of the canal and the cutting process induce a martensitic transformation with a reversible strain increasing the flexibility of the instrument. However, this flexibility can be insufficient to correctly follow the severely curved canals leading to premature instrumental fracture [1,25,26]. The increase of transformation temperatures (M_s_, M_f_, A_s_ and A_f_) of NiTi results in a more martensite phase at clinically relevant temperatures [25,27,28], which makes heat-treated NiTi instruments more flexible and more resistant to fatigue than conventional NiTi instruments.

There are many scientific publications about the mechanical properties of heat-treated NiTi files, especially about their bending and torsional resistance and cyclic fatigue [29,30,31,32,33,34,35]. To evaluate the flexibility of heat-treated endodontic files, the majority of studies compared heat-treated files to non-heat-treated ones during bending and torsion tests. Some of them compared instruments from the same brand with the same geometry and design, under different heat treatment conditions [9,27,29]. These studies have shown that heat-treated endodontic files have higher flexibility compared to conventional NiTi instruments [9,27,29]. Other studies have compared endodontic files from different brands with various geometrical designs, different operative motions, and heat treatment technologies [33,34,35]. These studies have demonstrated that many factors can influence mechanical resistance. Heat treatment, different cross-sectional designs, tip and taper dimensions, pitch lengths, and operative motions could affect the resistance to both the normal and shear stresses induced by bending and torsion [1,36].

Heat treatment processes for endodontic files are generally not disclosed by manufacturers who apply their own protocols often protected by industrial secrecy [13]. Manufacturers constantly need to improve their processes to offer instruments with better mechanical performances, especially in terms of flexibility. In fact, the flexibility of NiTi instruments plays a crucial role in successful endodontic treatment, especially in highly curved canals. It allows suitable canal enlargement while preserving the instrument in a central position within the canal, leading to minimal undesirable change in the shape of curved canals [5].

Some studies investigated the influence of heat treatment temperature on the flexibility of conventional endodontic instruments by applying different heat treatments. Kuhn and Jordan (2002) located the critical temperature at 600 °C. Heat treatment below 600 °C generally increases flexibility, while treatment above 600 °C increases stiffness. Other studies investigated the influence of heat treatment parameters such as temperature and quenching on the mechanical properties of non-machined NiTi alloy [17]. Yahata et al. (2009) investigated the effect of heat treatment on the bending properties of NiTi endodontic instruments by testing conical specimens (0.30 mm tip diameter and 0.06 taper) [22]. They concluded that in the elastic and superelastic ranges, the 440 °C—30 min specimens displayed the highest flexibility in bending. They demonstrated that the transformation temperature was increased by heat treatment in a temperature-dependent manner, while heat treatment time has less influence on flexibility [22]. Miyara et al. (2014) found that flexibility (tested in the superelastic range) is increased with heat treatments at 400, 450, or 500 °C compared to a heat treatment at 300 °C or non-heat-treated samples [24].

Therefore, it is important to better understand how heat treatment influences the flexibility of NiTi wires used for the machining of endodontic instruments, without taking into account geometrical parameters. The obtained results would only show the effect of heat-treatment parameters for a constant circular section wire and get rid of the effect of the complex geometry of endodontic files on stress distribution during bending tests.

In order to figure out the single and interaction effects of heat treatment factors on flexibility, the use of statistical methods devoted to the Design of Experiments (DoE) is an appropriate solution to provide useful empirical models for designing new instruments or for optimizing manufacturing processes. The Design of Experiments (DoE), initially proposed by Fisher [37,38], consists of determining a parsimonious set of multifactorial tests to be carried out to collect the data needed to answer a specific question with acceptable precision. The type of question—e.g., factor screening, interaction analysis, response optimization, robustness studies—determines the appropriate choice of DoE [39]. DoE is now included in reputable development practices such as the Quality by Design approach [40] to design new materials used in pharmaceuticals [41] or medical devices [42].

Two studies were conducted in the field of endodontics using DoE. The first one aimed to investigate and optimize the main factors influencing the forces and moments induced by the penetration/removal motion and to establish an efficient predictive model for root canal preparation [43]. The second one aimed to find optimized root canal angle and speed of rotation giving maximum fatigue life of the endodontic file [44].

Therefore, the purpose of this study is to investigate through a full factorial DoE the influence of three parameters of heat treatment on the flexibility of NiTi wire: heating temperature, holding time, and cooling method. Interactions between these parameters will also be investigated. Flexibility will be evaluated by measurement of torque for a given maximum rotation, through a bending test under ISO 3630-1 standard [45]. Contrary to previous studies, the bending test will be carried out at fixed number of cycles by using an adapted designed bending and torsion bench [46].

## 2. Materials and Methods

### 2.1. Sample Selection

The investigated material was a commercial polycrystalline NiTi SMA wire provided by Fort Wayne Metal (Fort Wayne Metal, Fort Wayne, IN, USA) with a composition of nearly 50:50 atomic %. The wire has a 1 mm diameter and corresponds to the one routinely used for machining endodontic instruments and prototypes.

NiTi samples were cut to a 25 mm length from the same batch to prevent the scattering effect induced by processing variations such as (i) transformation temperatures before processing, (ii) chemical composition (percentage of Nickel and Titanium), and (iii) cold work percentage. This length allows us to correctly clamp both sides of the wire in order to perform reproducible bending tests while respecting the guidelines of the ISO 3630-1 standard [45]. Samples were divided into two groups: one group without heat treatment (control group) and one heat-treated group at different temperatures and different holding times.

### 2.2. Statistical Design of Experiment

A full factorial DoE was used to figure out the signal and interaction effects of heat treatment parameters on the bending response of the NiTi wire. Temperature, holding time, and cooling method were selected as contributing factors. Three levels of heating temperature and holding times, and two levels of cooling method were considered (water quenching and air cooling). Two interactions were retained, which are temperature/holding time and temperature/cooling method. These choices were established after preliminary tests based on a Once-Factor-At-A-Time method, in which heat treatments were performed with three levels for each factor applied separately. In addition to air and water, in-furnace cooling was the third level. These tests showed that bending and tensile responses are slightly influenced by cooling method. Thus, only air and water cooling were selected. The levels of the contributing factors in this DoE are presented in Table 1.

A full factorial design, composed of 18 experimental trials (using all the possible combinations of factors and levels) was implemented. Each experiment was carried out in triplicate to better estimate the single and interaction effects.

The output responses (Y) measured during the experiments are:

− Y1: Maximum bending moment: the value of the torque (bending moment) when the bending rotation is maximum (45°) during the last loading cycle (bending tests are described later in this document).
− Y2: The size of the hysteresis: the difference between the bending moment measured during loading and unloading of the last cycle at two thirds of the maximum applied rotation (45°).
− Y3: Stiffness during martensitic transformation (transformation slope): the slope coefficient of the transformation domain in the moment-rotation diagram, chosen between a first point positioned at one third of maximum rotation and a second point positioned at two thirds of maximum rotation.

### 2.3. Heat Treatment

**Figure 1:**
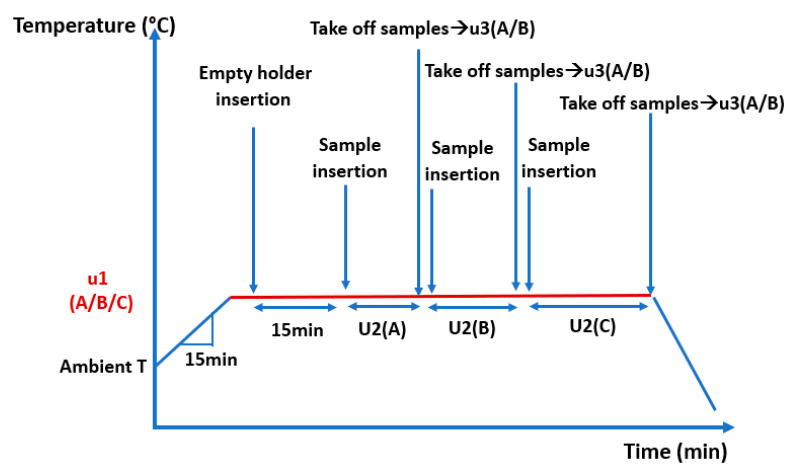
Heat treatment protocol.

Heat treatment was performed using a Naberthem LHT 01/17 D furnace (Naberthem GmbH, Lilienthal, Germany). It allows us to carry out tests at temperatures up to 1650 °C. The furnace is controlled by the “Nabertherm VCD” software v 01.28. Samples were placed in a stainless-steel metal holder to withstand high temperatures. After reaching and stabilizing the target temperature (325 °C for example), the stainless-steel holder was placed empty in the furnace and heated before introducing the samples in order to ensure that all samples were heated in the same conditions. When the desired temperature is reached, the heated holder is filled with samples and inserted in the oven for a duration of 15 min (first group of samples). After being taken off for cooling of the samples, the holder is filled with the second group of samples (heat treatment for 30 min) and then taken off for cooling. Finally, the holder is filled again with the third group of samples and then cooled after 45 min of heat treatment. This procedure is repeated for the two other temperatures (375 °C and 425 °C) (Figure 1).

### 2.4. Bending Test

**Figure 2:**
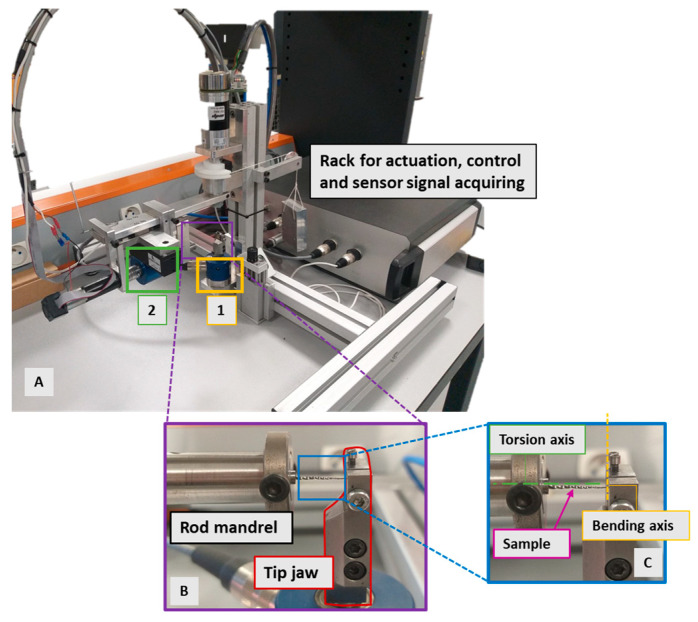
(**A**) Testing device, 1. bending sensor; 2. torsion sensor. (**B**,**C**) Sub-systems for sample clamping and positioning.

The bending test was carried out at room temperature (20 °C) using a testing machine developed by Xolin et al. [46]. It allows us to apply a bending and/or torsion rotation to a sample or a wire clamped in the two extremities leaving the axial displacement free. This testing device consists of three sub-systems with two perpendicular axes. The first sub-system controls the torsion loading and is parallel to the sample axis. It consists of a direct current (DC) engine as an actuator to apply the torsion rotation, a sensor to measure the induced moment, and two connections: a clamped connection to the sample and a sliding one with the bending sub-system (Figure 2). This latter consists of one DC engine as an actuator to apply the bending rotation, a sensor to measure the induced bending moment, and a rotation connection to the device frame. This sub-system has a rotation axis perpendicular and coplanar to the sample axis. The third sub-system is the frame of the device on which the bending sensor and actuator are positioned [46]. This device allows for controlling the torsion and the bending rotations in a separate or a combined way. The bending and torsion torque sensors have a measurement range of ±500 and ±200 N.mm, respectively.

**Figure 3:**
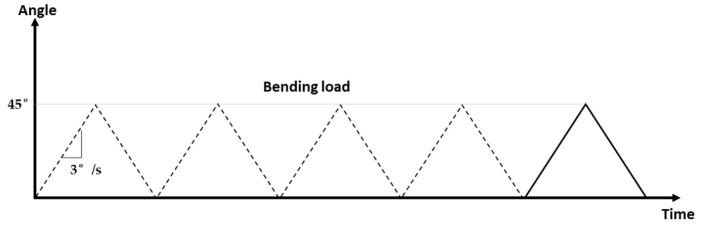
Simple bending load. In this study, θ = 45°, loading-unloading cycles = 5, rotation speed ω = 3°/s.

The bending test is performed according to the ISO 3630-1 standard [45]. Every sample is held by its rod using the chuck positioned on the torsion bearing and is held at the tip on its first 3 mm by the jaws positioned on the bending torque sensor. Standard ISO 3630-1 recommends using a brass piece to clamp the tip of the sample in order to limit overloading [45]. These brass pieces are locally deformed and replaced when their surface is printed by tested samples. The wire samples were subjected to five loading/unloading cycles at 45° rotation with a speed of 3°/s (Figure 3).

### 2.5. Statistical Analysis

The first step was to explore and visualize the data using an exploratory analysis followed by a comparison between several models, considering different effects in each model, to determine the most critical effects.

Firstly, several investigation models were adjusted. The studied models are the following:

− the complete model (u1, u2, u3, u1*u2, u1*u3, u2*u3, u1*u2*u3). This model is the complete linear one with simple factor effects, quadratic effects, and interaction effects up to order 3.
− The following sub-models were applied:
− model with simple effects, and interaction effects of order 2 (u1, u2, u3, u1*u2, u1*u3 and u2*u3);
− model with only the simple effects (u1, u2, u3);
− complete model by removing u3 (u1, u2, u1*u2);
− complete model by removing u2 (u1, u3, u1*u3);
− complete model by removing u1 (u2, u3, u2*u3);
− model with only the effect of u1 (u1);
− model with only the effect of u2 (u2);
− model with only the effect of u3 (u3).

Then, for each model, two indices of quality were computed: the R-square (R^2^) and the adjusted R^2^. The R^2^ represents the variability of the model response and indicates the effectiveness of the developed model. It is always between 0 and 1 and a value of R-square equal to 1 indicates 100% effectiveness of the model. All statistical analyses were performed using R 4.1.0 software.

## 3. Results

### 3.1. Exploratory Analysis

**Figure 4:**
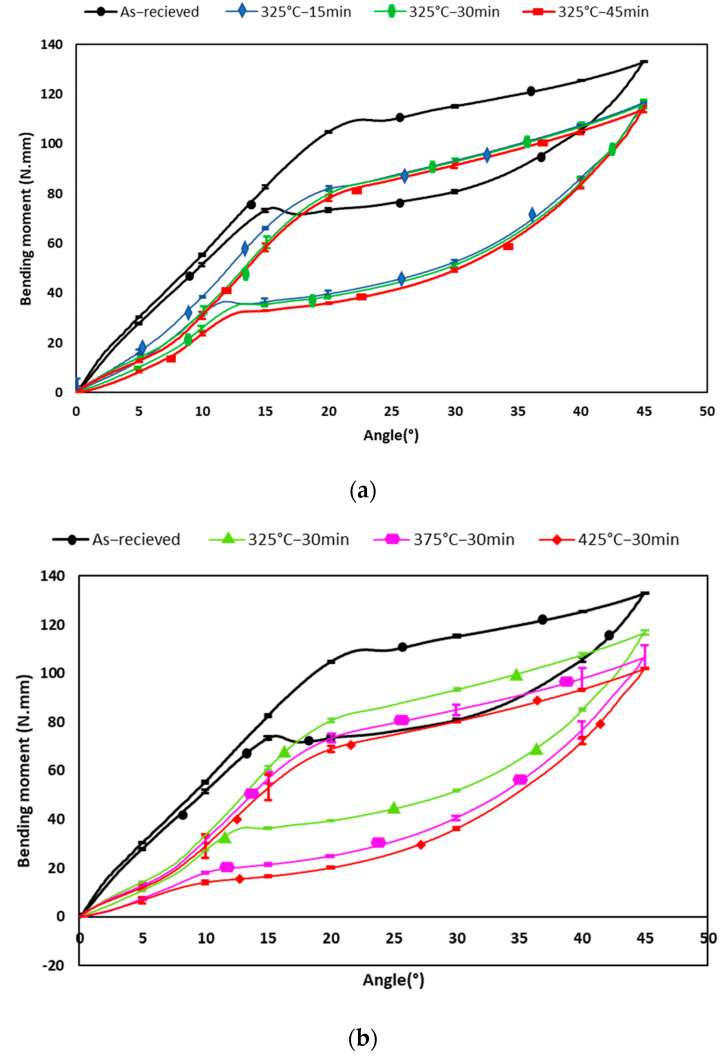
Response of heat treated NiTi wires. (**a**) Heat−treatment at 325 °C with different holding times and water quenched. (**b**) Heat treatment with different temperatures for 30 min and air cooled. The black curve shows the non-heat-treated wire (control group).

Figure 4 shows the average responses of a NiTi wire with different heat treatments under bending loading.

On the application of bending loading as described in the materials and methods section, the corresponding initial portion of moment-rotation curve shows, for the non-heat-treated wires, a linear relationship corresponding to the elastic behavior of the SMA at the austenitic state. However, for heat-treated wires, the line is curved with a lower elastic slope. The occurring of R-phase during this step induces this curvature. The corresponding inelastic strain remains small and has no significant influence on the resulting flexibility. Above this range, the bending moment level becomes almost constant owing to the stress-induced martensitic transformation. During the unloading process, the bending moment decreases and becomes constant owing to the reverse martensitic transformation.

Subsequently, elastic unloading occurs without residual plastic strain (Figure 4a,b). A hysteretic response is observed between loading and unloading during the forward and reverse martensitic transformation steps. The bending moment and rotation curves show that heat-treated wires are more flexible than the received wires (control group), with lower values of maximum bending moment and larger hysteresis.

**Figure 5:**
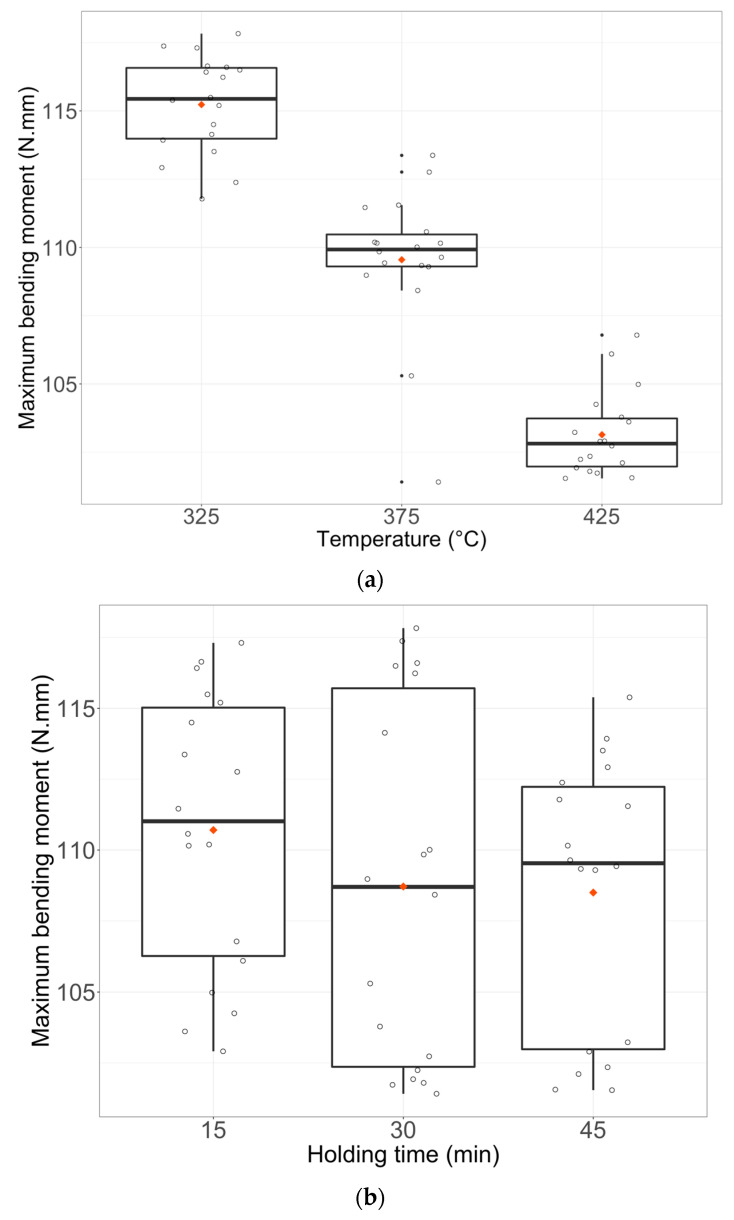

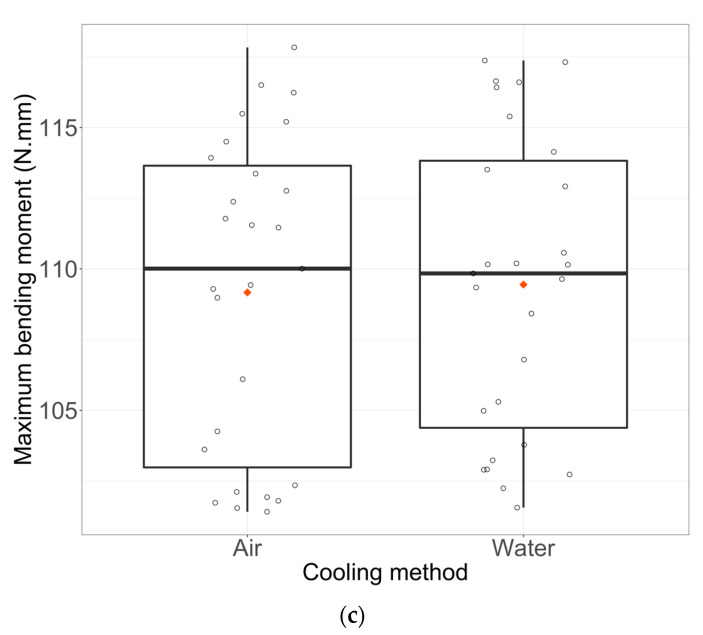
Boxplots of Y1 (Maximum bending moment) with the simple effect of factors u1 (**a**), u2 (**b**), and u3 (**c**). Each boxplot plotted in black shows the median and quartiles (rectangle widths stand for 25% and 75% quartiles) and points in dark stand for extreme values. Data points are shown and the mean is represented by a red lozenge.

**Figure 6:**
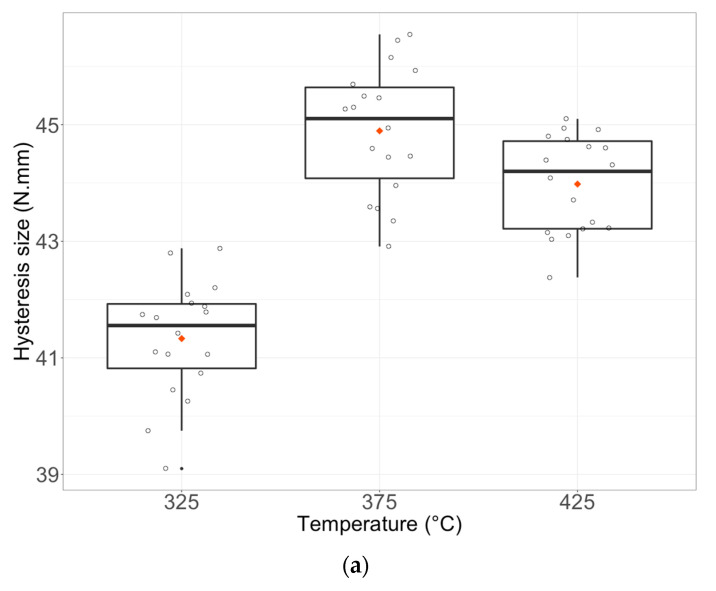

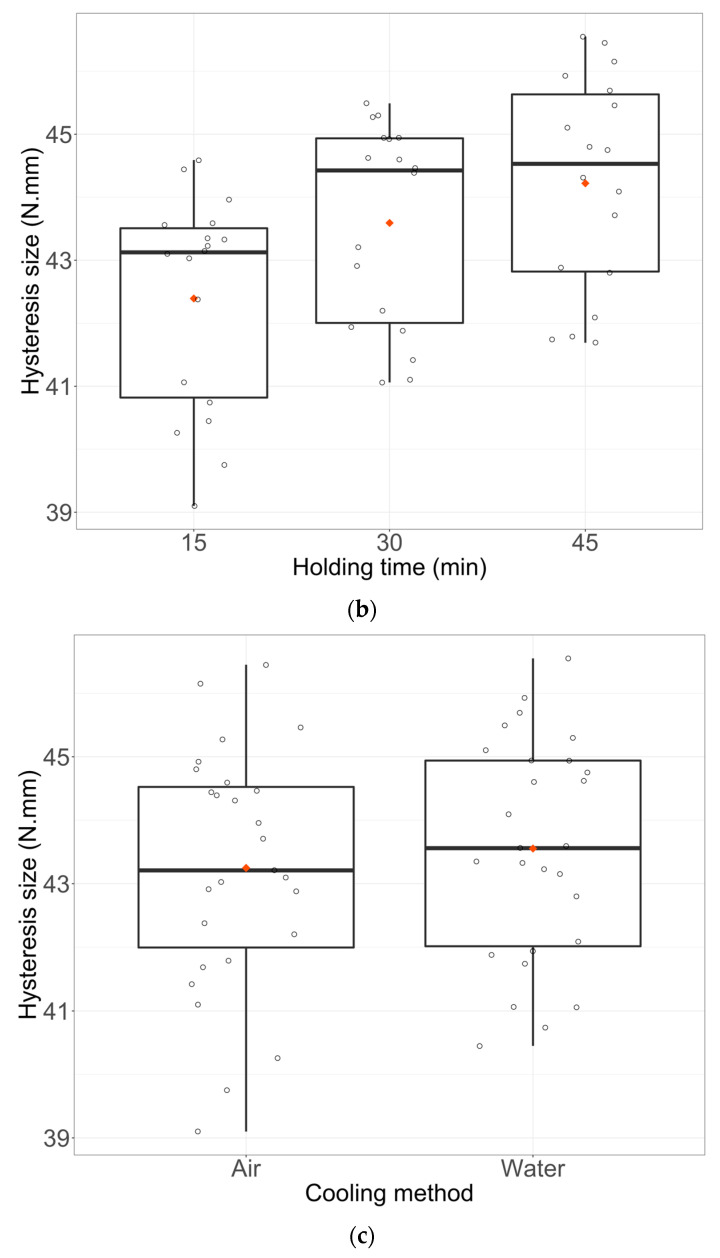
Boxplots of Y2 (Hysteresis size) with the simple effect of factor u1 (**a**), u2 (**b**), and u3 (**c**). Each boxplot plotted in black shows the median and quartiles (rectangle widths stand for 25% and 75% quartiles) and points in dark stand for extreme values. Data points are shown and the mean is represented by a red lozenge.

**Figure 7:**
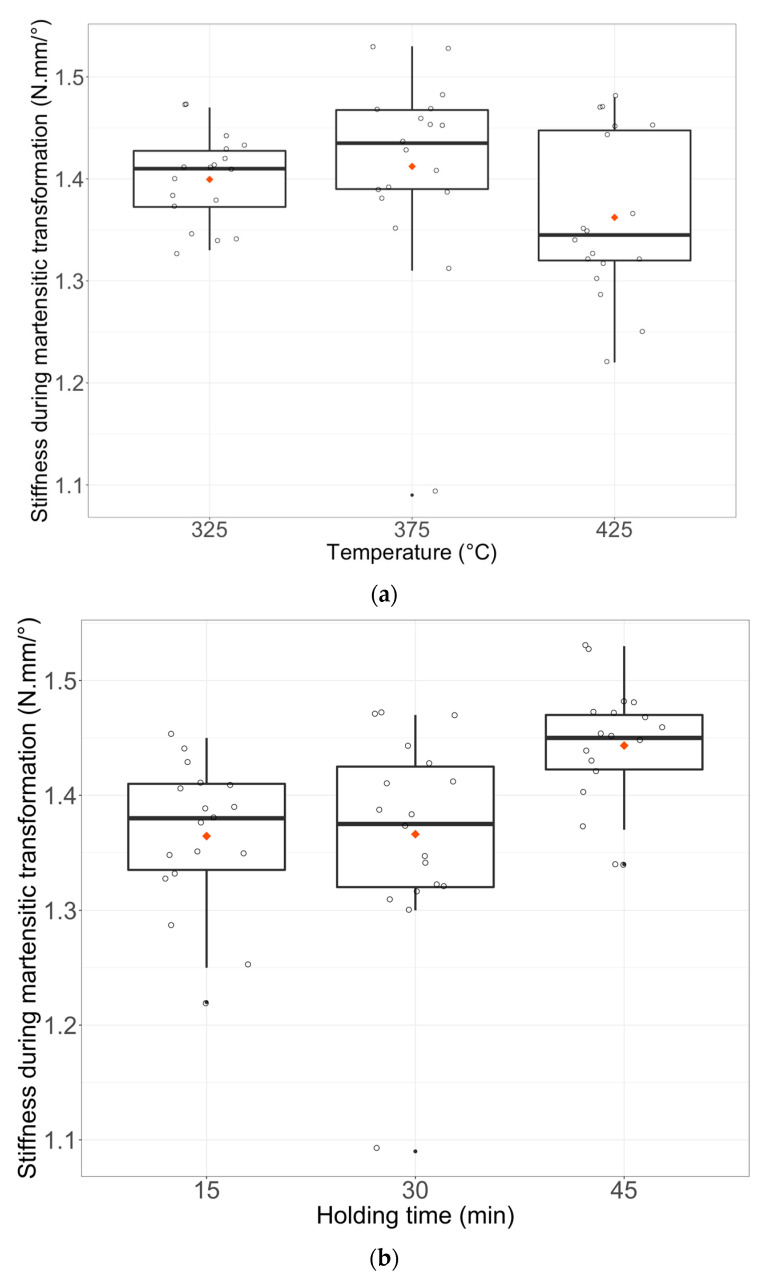

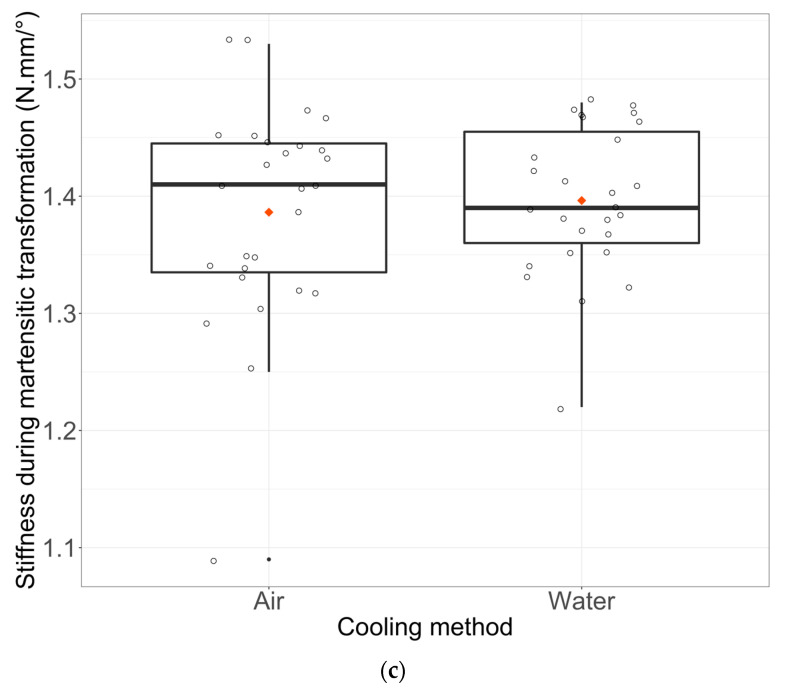
Boxplots of Y3 (Stiffness during martensitic transformation) with the simple effect of factor u1 (**a**), u2 (**b**), and u3 (**c**). Each boxplot plotted in black shows the median and quartiles (rectangle widths stand for 25% and 75% quartiles) and points in dark stand for extreme values. Data points are shown and the mean is represented by a red lozenge.

Boxplots of Y1 (maximum bending moment), Y2 (hysteresis size), and Y3 (transformation slope) in Figure 5, Figure 6 and Figure 7 show the raw data for each modality with median and quartiles. The increase of heat-treatment temperatures leads to the increase of the NiTi wires flexibility by decreasing the maximum bending moment (Response Y1). Wires heat-treated for 30 min showed the highest flexibility while wires heat-treated for 15 min showed the lowest flexibility. Finally, wires cooled in air have the same flexibility as the quenched ones (Figure 5a–c).

Wires heated at 325 °C had the smallest hysteresis (response Y2). The hysteresis size increases at 375 °C to decrease again at 425°. Wires heat-treated for 15 min showed the smallest hysteresis while those heat-treated during 30 and 45 min showed the largest hysteresis. Finally, the hysteresis size is the same for both air- and water-cooled wires (Figure 6a–c).

The lowest stiffness during martensitic transformation (Response Y3) was for wires heated at 425 °C. However, wires heat-treated for 45 min showed the highest stiffness during transformation (Figure 7a–c).

**Figure 8:**
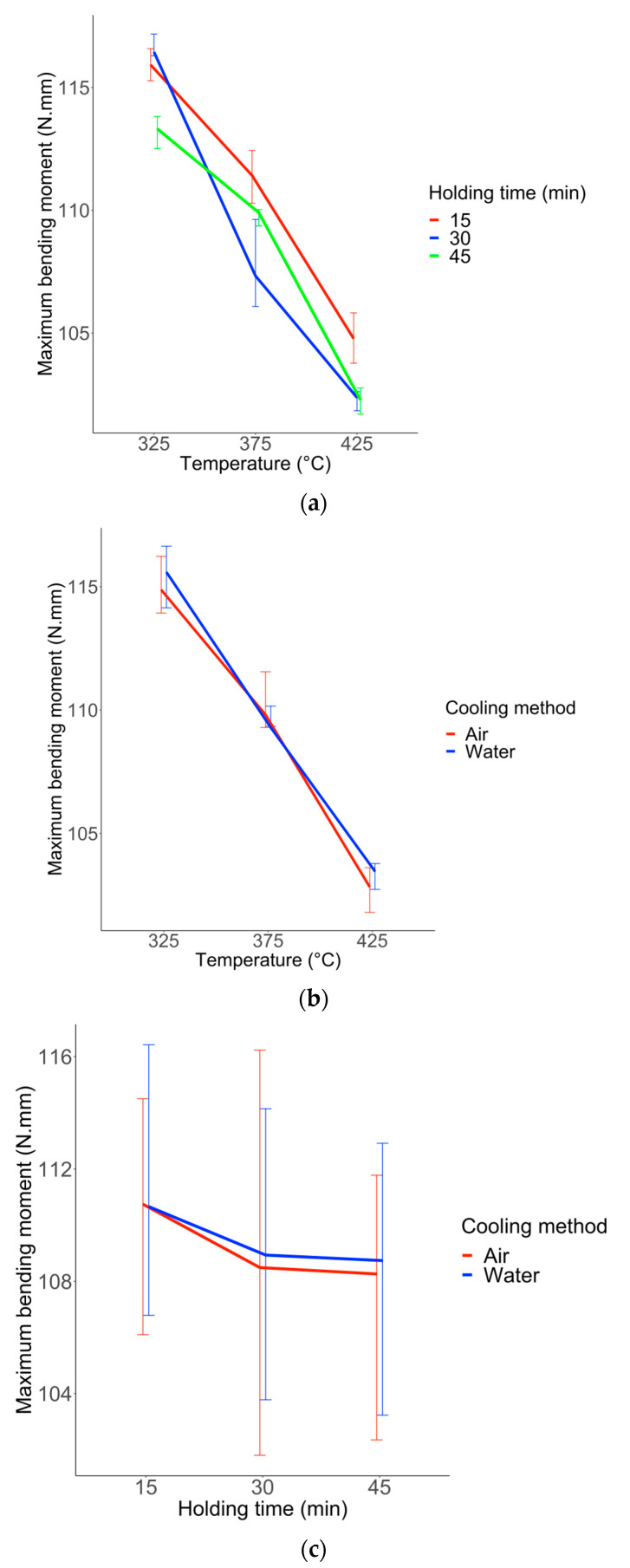
Interaction plot for each pair of factors for response Y1. (**a**) interaction u1 and u2 (temperature and holding time). (**b**) interaction u1 and u3 (temperature and cooling method). (**c**) interaction u2 and u3 (holding time and cooling method). The extremities of the associated vertical line stand for 25% and 75% quartiles.

**Figure 9:**
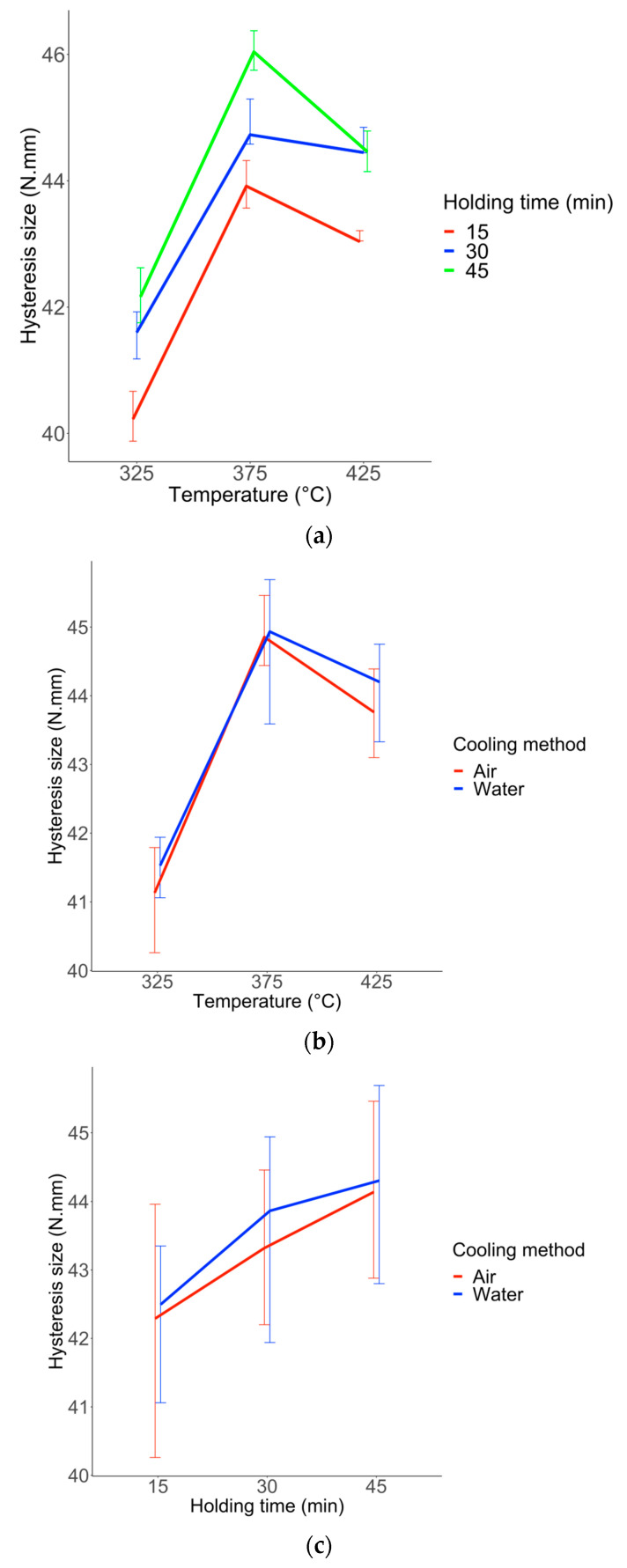
Interaction plot for each pair of factors for response Y2. (**a**) interaction u1 and u2 (temperature and holding time). (**b**) interaction u1 and u3 (temperature and cooling method). (**c**) interaction u2 and u3 (holding time and cooling method). Each point represents the mean of responses Y2 for a combination of two modalities. The extremities of the associated vertical line stand for 25% and 75% quartiles.

**Figure 10:**
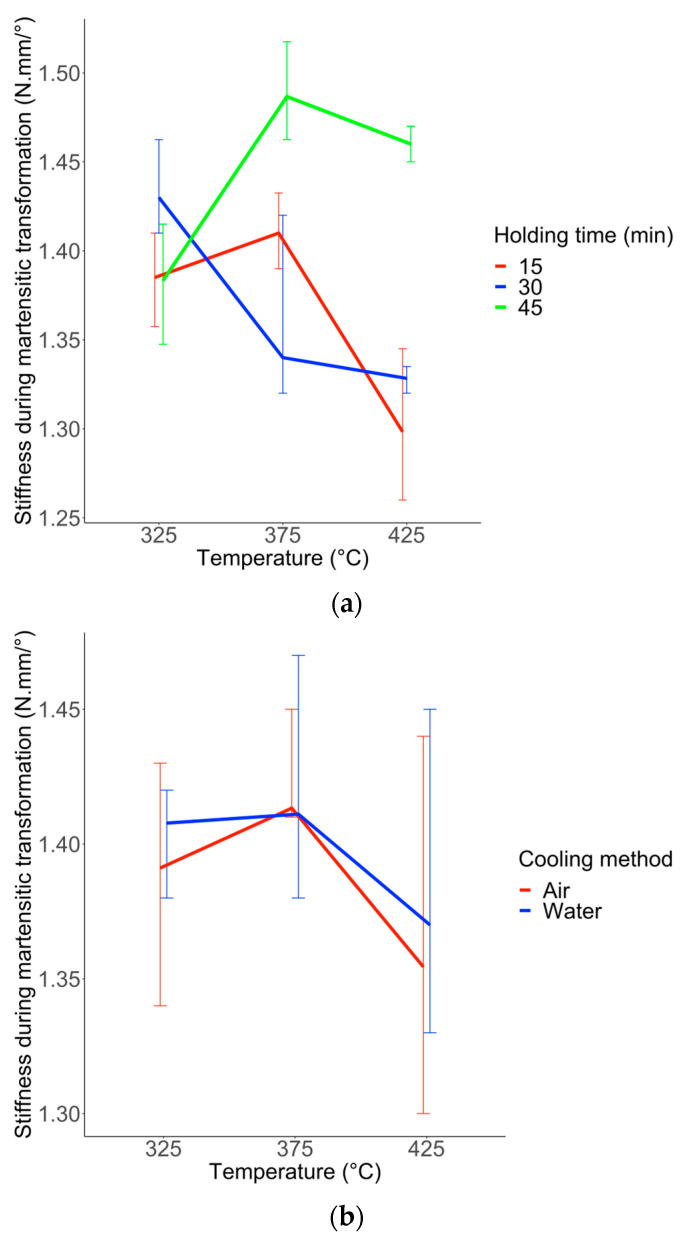

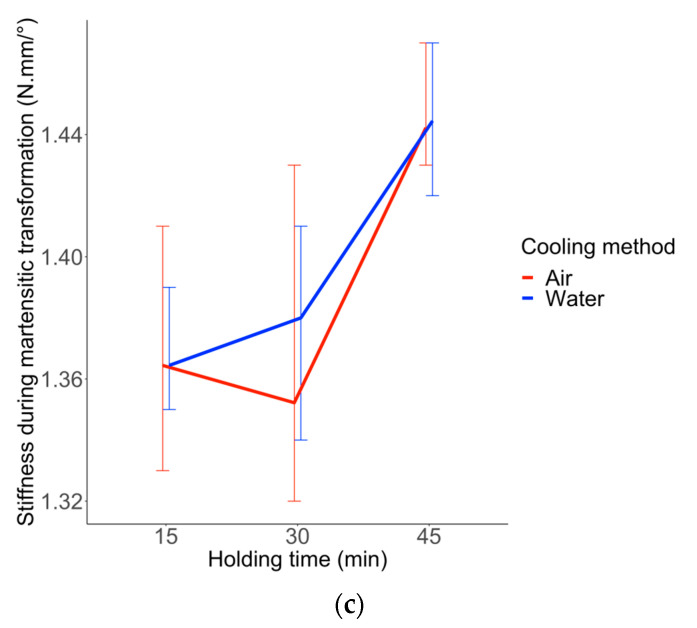
Interaction plot for each pair of factors for response Y3. (**a**) interaction u1 and u2 (temperature and holding time). (**b**) interaction u1 and u3 (temperature and cooling method). (**c**) interaction u2 and u3 (holding time and cooling method). The extremities of the associated vertical line stand for 25% and 75% quartiles.

Plots in Figure 8, Figure 9 and Figure 10 visualize the potential order of two interaction effects for every response (Y1, Y2, and Y3). Higher order interactions are considered to be negligible. It can be assumed that there is an interaction effect when the profiles are not parallel.

Table 2 shows the identified interactions between factors for Y1 (Maximum bending moment), Y2 (Hysteresis size), and Y3 (Transformation slope) responses. The only interaction which has a significant impact on the wire response during bending tests is between temperature and holding time.

### 3.2. Statistical Analysis

Table 3, Table 4 and Table 5 show the quality of the models based on the R-square and adjusted R-square.

Based on these results, we concluded that:

− For Y1 (Maximum bending moment): the only interaction that exists in this DoE is between temperature and holding time. *u1* (temperature in °C) is the most critical factor for Y1 (N.mm). It explains more than 85% of the variability of the response without even considering the interaction effect with *u2* (holding time in min). This R^2^ of more than 85% shows that the model is effective at predicting the relationship between contributing factors and the response Y1. *u2* also has an impact on Y1 through the interaction effect with *u1*. Finally, the cooling method (*u3*) is not a critical factor for Y1.
− For Y2 (Hysteresis size): the simple effect of *u1* is critical for Y2 followed by the simple effect of *u2*. More than 71% of the variability is explained only by the effect of *u1* and almost 18% by the simple effect of *u2*. The other effects are not significant.
− For Y3 (Stiffness during martensitic transformation): the complete model explains 62.5% of the variability for Y3. The most critical effect is the interaction effect between *u1*, *u2,* and Y3. There is also a simple effect of *u2*. In fact, looking at the R^2^ of model 4, we can see that 57% of the variability comes from simple and interaction effects of *u1* and *u2*. The third factor *u3* is not significant.

## 4. Discussion

The current study showed that the flexibility of NiTi wires was increased by heat treatment, and specifically by the heat treatment temperature. This latter is considered, within the study conditions, as the most critical parameter followed by the holding time.

To evaluate the bending properties of endodontic instruments, the ISO 3630-1 established a bending test, which involves clamping 3 mm of the tip of each instrument into a chuck and applying an angular deflection of 45° [5,45]. In this study, the bending test for NiTi samples was performed according to this standard by using a new bending-torsion testing device [46]. The curves obtained from the bending test (Figure 4a, b) show bending loads at specific angles of rotation, corresponding to two different ranges: the elastic and the superelastic ranges, respectively. The superelasticity of NiTi alloy is induced by martensitic transformation at constant temperature. The bending loading in the superelastic range is dependent on the yield stress required to induce martensitic transformation as predicted by the Clausius–Clapeyron relationship [22,47]. Low bending moments are indicative of high flexibility.

In both elastic and superelastic ranges, all heat treatment groups showed lower maximum bending moment values compared to the non-heat-treated group. This could be explained by the fact that the heat treatment reduces the crystalline defects in the microstructure and thus the complete martensitic transformation requires lower stress due to the increase of martensite interfaces mobility [17,22]. In elastic range, a deflection occurs at 5° of bending rotation (Figure 4) during loading and unloading. The observed stiffness is lower before the deflection and higher after. This response would be induced by R-phase, an intermediate phase between austenite and martensite, which competes with the transformation of the martensite phase. It occurs in cold worked NiTi alloys having a high density of dislocations, in aged nickel-rich NiTi alloys (Ti_3_Ni_4_ precipitations), or in certain ternary alloys [48,49]. Zhang and Sehitoglu showed through tensile tests that this response is characteristic of the R-phase in NiTi [50]. However, the lower apparent Young’s modulus of heat-treated wires in this elastic-range can be attributed to the presence of a certain percentage of R-phase in addition to austenite. In fact, the Young’s modulus of R-phase is lower than austenite at ambient temperature [1]. This intermediate transformation, even if it has an influence on the apparent elastic slope, has no impact on the maximum moment and therefore on the flexibility of the instrument. Its effect has therefore no influence during root canal preparation.

In superelastic range, the 425 °C heat-treated specimens revealed significantly lower maximum bending moment values than the 325 °C and 375 °C heat-treated samples and control specimens. In addition, the control specimens exhibited the highest maximum bending moment value of all the groups. Near-equiatomic NiTi SMAs contain three microstructural phases which are austenite, martensite, and R-phase. The relative proportions and characteristics of each phase determine the mechanical properties of SMAs [15,21,25]. Conventional superelastic NiTi SMAs have an austenite structure at room temperature and clinical use. However, when the transformation temperatures change by heat treatment on NiTi SMAs, martensite, R-phase, or more than one phase can be present at room temperature [21]. With the leaded microstructure, the R-phase occurs firstly during bending loading induced by root canal preparation. It induces a slight decrease of the apparent elastic slope of austenite. The martensitic transformation occurs for higher loading levels leading to a much more important transformation strain, increasing the flexibility of the instrument [48]. The R-phase has a low influence on the requested flexibility of the instrument.

The high correlation found in the present study between the maximum bending moment and the heat treatment temperature is an indication that temperature parameters are the most important ones affecting the flexibility of NiTi wires. Moreover, the 30 min and 45 min-treated samples showed greater flexibility than the 15 min-treated ones, but 30 min-treated samples are slightly more flexible than 45 min-treated ones. This result suggests that increasing the heat treatment holding time leads to the decrease of the wire bending load only up to a certain holding time. Similar observations are made regarding the transformation slope. In fact, samples heat treated at 425 °C showed the lowest slope and 45 min-treated samples presented the highest slope. These findings are in agreement with previous studies, despite differences in experimental conditions [22,24]. Yahata et al. [22] compared a control group with four other groups: heat-treatment at 440 °C or 500 °C, and heat treatment times of 10 or 30 min. They observed that the sample heated at 440 °C for 30 min had the lowest maximum bending moment value, both in the elastic (0.5 mm deflection) and superelastic (2 mm deflection) ranges through a cantilever-bending test [22]. Miyara et al. [24] only tested flexibility in the superelastic range (at 2 mm) and found that it is increased with a heat treatment at 400 °C, 450 °C, or 500 °C compared to a heat treatment at 300 °C or to the control group.

Proprietary methods of thermomechanical treatment of NiTi wires were developed by manufacturers and the first thermomechanically treated NiTi SMAs in dentistry were M-Wire^®^ (Memory-Wire), R phase™ heat treatment, and CM-Wire ™(Controlled-Memory-Wire). These three different types of wires are produced depending on the thermal treatment of the wires prior to or during manufacturing. Those different procedures have been developed to obtain superelastic wire blanks that maintain the stable martensite phase under clinical conditions [51]. Since then, some manufacturers have developed new files from M-wires with additional post-machining heat treatment such as the Gold-wire or the Blue-wire. Others have proposed different technologies of post-machining heat treatment such as T-wire, C-wire, and Max-wire.

Several studies compared the flexibility of these various heat-treated instruments. Unlike the present study, the majority of published studies compared the flexibility of marketed heat-treated endodontic files to non-heat-treated ones. Some of them compared instruments from the same brand with the same geometry and design, the only difference being the heat treatment. Others compared endodontic files from different brands with various geometrical designs, different operative motions, and different heat treatment technologies.

Despite the lack of information about the applied heat treatment, the NiTi files made of M-wire, R-phase heat treatment, and controlled memory wire have shown improved flexibility in comparison with conventional NiTi files. Pereira et al. (2015) reported that the flexibility was significantly higher for Typhoon (CM-wire) followed by Vortex Blue (Blue-wire), Profile Vortex (M-wire), and ProTaper Universal instruments (conventional superelastic NiTi wire) [52]. Elsaka et al. found that the WaveOne Gold (Gold heat treatment technology) had a significantly higher flexibility than Reciproc (M-wire technology) and Twisted File Adaptive instruments (R-phase technology) and that Reciproc instruments had a significantly lower flexibility than Twisted File Adaptive. [53]. Hou et al. (2020) compared, using a cantilever bending test, the flexibility of Blue and Gold heat-treated files. The results showed that the bending load values of WaveOne Gold and ProTaper Gold in the superelastic range were higher than those of Reciproc Blue instruments but lower than ProTaper Next, WaveOne, and ProTaper. Thus, after Gold and Blue heat treatment, NiTi instruments presented significantly better bending flexibility and cyclic fatigue resistance, in comparison with M-wire and conventional instruments [9]. Goo et al. (2017) concluded, by comparing V-Taper 2, V-Taper 2H, Hyflex CM, HyFlex EDM and ProTaper Next X2, that CM-wire instruments showed higher flexibility than M-wire and conventional NiTi instruments [32]. It is worth noting that the compared instruments have different geometrical designs (different cross-section, taper) or are made using different manufacturing methods which can also influence their response during bending tests and this should be taken into account.

During a bending test, a hysteresis loop is formed due to the difference between the loading and unloading stress plateaus where energy is dissipated. This hysteresis is associated with internal friction effects [54,55]. In endodontics, where superelasticity is exploited (material can be loaded to provide large, recoverable strains at relatively constant stress levels), instruments with smaller hysteresis loops are preferable [56,57]. A narrower stress hysteresis means that more austenite can be transformed during the stress-induced martensitic transformation [58]. In addition, a low hysteresis leads to a better mechanical performance (optimal pseudo-elastic behavior) and therefore to an optimum lifespan of the endodontic instrument [55,59,60].

In the current study, hysteresis size is influenced mostly by heat treatment temperature followed by holding time. Increasing the heat treatment temperature and the holding time increases the hysteresis size up to a certain point. This study shows that heating at 375 °C or heating for 45 min leads to the largest hysteresis. Although many studies addressing the effect of heat treatment on superelastic behavior and shape memory effect have been published, few papers focus on the influence of heat treatment on the area and energy dissipation of the hysteresis loop of NiTi alloys [61]. Oliveira et al. evaluated quantitatively how aging at different temperatures and times can change the hysteresis loop in a superelastic regime of Ni (50.7%)-Ti tension springs. The study concluded that the energy dissipated per cycle increases with aging time [61].

Finally, all the samples cooled in water and in air showed a similar bending response, hysteresis size, and stiffness during martensitic transformation. This result suggests that the cooling method may only slightly affect the bending properties of NiTi wires. Through a full factorial DoE, possible interaction between parameters was assessed and only the temperature-holding time had an impact on the bending load and stiffness during martensitic transformation. This interaction does not impact on the hysteresis size of the tested wires.

Regression analysis was performed to estimate the relationship between contributing and output variables to assess the validity of the developed model. A value of R-square equal to 1 indicates 100% effectiveness of the model. In our study, the R-square is more than 90%, which shows that the model is effective at predicting the relationship between contributing factors (temperature, holding time, and cooling method), flexibility, and hysteresis size. However, the model is less effective at predicting stiffness during transformation with an R-square below 65%.

The use of the DoE has allowed us to generate data that could not be obtained with a classical experimentation (without DoE). These results helped to identify the most important factors to manufacture instruments that are clinically more efficient in terms of mechanical performance. The use of DoE in the field of endodontics is uncommon with only a few papers published [12,44,46]. This work is a key step towards more studies using DoE to investigate other parameters or other mechanical properties of NiTi alloys used in endodontics.

## 5. Conclusions

Bending properties of NiTi alloys are influenced by several factors, including heat-treatment. This study showed that heat treatment has an important impact on the NiTi wire response during bending tests and may be effective at increasing its flexibility. It appears that heat treatment temperature is the most critical factor influencing the flexibility and hysteresis size of the NiTi wire followed by the holding time, while the cooling method has a negligible effect. The full factorial DoE helped to find the optimized parameters which give maximum flexibility: 425 °C of heat temperature with a holding time of 30 min. Regression analysis confirms the effectiveness of heat treatment temperature and holding time on flexibility and hysteresis size of NiTi SMAs.

The flexibility of NiTi endodontic instruments is influenced not only by the heat treatment but also by its geometrical parameters. Thus, to understand this complex interaction of multiple parameters, further studies will be performed using DoE on NiTi prototype instruments with a given design.

## Figures and Tables

**Table 1:** Contributing factors and their levels.

Factors	Levels		
	A	B	C
**Temperature [°C]**	325	375	425
**Holding time [min]**	15	30	45
**Cooling method**	Air	Water	/

**Table 2:** Interaction between factors.

Factors	Interaction u1/u2	Interaction u1/u3	Interaction u2/u3
Y1 (Maximum bending moment)	Yes	No	No
Y2 (Hysteresis size)	No	No	No
Y3 (Transformation slope)	Yes	No	No

**Table 3:** Quality of the models of Y1 (Maximum bending moment). u1. temperature, u2. holding time), and u3 (cooling method).

Model	R2(%)	Adjusted R2 (%)
Model 1 (u1 × u2 × u3)	94.24	91.51
Model 2 (u1 × u2 + u1 × u3 + u2 × u3)	93.37	91.22
Model 3 (u1 + u2 + u3)	89.4	88.3
Model 4 (u1 × u2)	92.97	91.72
Model 5 (u1 × u3)	86.21	84.77
Model 6 (u2 × u3)	3.6	−6.44
Model 7 (u1)	85.86	85.3
Model 8 (u2)	3.47	−0.31
Model 9 (u3)	0.07	−1.85

**Table 4:** Quality of the models of Y2 (Hysteresis size).

Model	R2(%)	Adjusted R2 (%)
Model 1 (u1 × u2 × u3)	93.82	90.9
Model 2 (u1 × u2 + u1 × u3 + u2 × u3)	91.97	89.36
Model 3 (u1 + u2 + u3)	90	88.96
Model 4 (u1 × u2)	90.82	89.19
Model 5 (u1 × u3)	72.25	69.36
Model 6 (u2 × u3)	18.91	10.46
Model 7 (u1)	71.32	70.19
Model 8 (u2)	17.96	14.74
Model 9 (u3)	0.73	−1.18

**Table 5:** Quality of the models of Y3 (Stiffness during martensitic transformation).

Model	R2 (%)	Adjusted R2 (%)
Model 1 (u1 × u2 × u3)	62.5	44.8
Model 2 (u1 × u2 + u1 × u3 + u2 × u3)	58.05	44.42
Model 3 (u1 + u2 + u3)	30.1	22.81
Model 4 (u1 × u2)	56.68	48.98
Model 5 (u1 × u3)	8.12	−1.45
Model 6 (u2 × u3)	23.35	15.36
Model 7 (u1)	7.4	3.77
Model 8 (u2)	22.28	19.24
Model 9 (u3)	0.41	−1.5

## Data Availability

Data is unavailable due to privacy restrictions.
